# Mit Kontaktdaten gegen die Pandemie: Zur Ethik von Corona Warn-Apps

**DOI:** 10.1007/s00481-021-00629-y

**Published:** 2021-04-30

**Authors:** Philippe van Basshuysen, Lucie White

**Affiliations:** Institut für Philosophie, Leibniz Universität Hannover, Im Moore 21, 30167 Hannover, Deutschland

**Keywords:** COVID-19, Öffentliche Gesundheit, Digitale Kontaktverfolgung, Corona-Warn-App, Datenschutz-Ethik, Wertekonflikt, COVID-19, Public health, Contact tracing, Corona apps, Privacy, Ethical conflicts

## Abstract

Zu Beginn der Pandemie im Frühjahr 2020, und nach einem weitreichenden Lockdown, ruhten große Erwartungen auf Corona-Warn-Apps, um einen erneuten Lockdown zu verhindern. Diese Erwartungen haben sich nicht erfüllt; stattdessen wurden in Deutschland als Reaktion auf erneute Wellen von COVID-19 weitere Kontaktbeschränkungen verordnet. Wie hätte die digitale Kontaktverfolgung wirksamer gestaltet werden können? Wir argumentieren, dass es ein Spannungsfeld zwischen der Datensparsamkeit und einer wirksamen Bekämpfung der Pandemie besteht. Im Gegensatz zur deutschen Corona-Warn-App wäre eine Variante der App, in der pseudonymisierte Kennungen zentral gespeichert werden, in der Lage gewesen, die Effektivität der Kontaktverfolgung entscheidend zu erhöhen. Schließlich argumentieren wir, dass das Spannungsfeld zwischen Datensparsamkeit und einer wirksamen Pandemiebekämpfung sich jedoch nicht in einen Wertekonflikt übersetzt, weil zentrale Systeme uns trotz ihrer erhöhten Wirksamkeit nicht vor deutlich gravierendere Probleme beim Datenschutz stellen als dezentrale Systeme. Zentrale Möglichkeiten der digitalen Kontaktverfolgung wären daher ethisch gerechtfertigt, um auf weitere Wellen von COVID-19 oder auf zukünftige Epidemien effektiv zu reagieren.

## Abstract (en)

**Definition of the problem:**

In spring 2020, as much of the world was emerging from widespread “lockdowns” as an emergency measure to combat the spread of SARS-CoV‑2, there was sustained discussion about how to lift measures while preventing further waves of the virus and the need for further lockdowns. One strategy that attracted significant attention was the use of digital contact-tracing apps to quickly alert users of possible exposure to the virus, and to direct them into quarantine. The initially high expectations placed upon this strategy were not met—despite the implementation of a digital contact-tracing app in Germany, further restrictions have been placed on the general population in response to further waves of the virus. We consider how digital contact tracing might have been made more effective.

**Arguments:**

We argue that there is a conflict between collecting as little data as possible, and more effective epidemic control. In contrast to the “Corona-Warn-App” that was implemented in Germany, an app that stored more information on a central server (a so-called “centralized” app) had the potential to significantly decrease viral spread. We then look at the privacy-based arguments against the centralized storage of information, suggesting that “decentralized” systems have privacy problems of their own.

**Results:**

The German debate on digital contact tracing apps was quickly dominated by privacy concerns, to the detriment of other ethical factors such as enhancing potential effectiveness. Furthermore, the potential problems with privacy inherent in decentralized apps were obscured in the discussion. Once we recognize these two aspects, we can see that there is an argument to be made for preferring centralized digital contact-tracing apps.

## Einleitung

Rückblick: In einem Gesetzesentwurf vom 20. März 2020 sollten dem Bundesministerium für Gesundheit sowie zuständigen Gesundheitsbehörden bei „Feststellung einer epidemischen Lage von nationaler Tragweite“ weitreichende Befugnisse eingeräumt werden, die Datenschützer alarmierten. In der Begründung des Gesetzesentwurfs wurde darauf hingewiesen, dass die Nachverfolgung von Standortdaten, wie z. B. in Südkorea praktiziert, dabei helfen könne, Kontaktpersonen von COVID-19 Patienten zu ermitteln, um Infektionsketten wirksam zu unterbrechen. Die zuständigen Gesundheitsbehörden sollten deshalb dazu befähigt werden, „Kontaktpersonen von erkrankten Personen anhand der Auswertung von Standortdaten des Mobilfunkgerätes zu ermitteln, dadurch die Bewegung von Personen zu verfolgen und im Verdachtsfall zu kontaktieren“ (Bundesgesundheitsministerium 2020, S. 20).

Nach massiver öffentlicher Kritik wurde dieser Passus gestrichen (Neuerer und Waschinski 2020). Stattdessen sollten Personen nun eine Corona-Warn-App freiwillig herunterladen können, die über Bluetooth-Signale mit anderen, in der Nähe befindlichen Mobiltelefonen Kennungen austauscht. Bei einer Infektion würden diese auf einen zentralen Server geladen und möglicherweise infizierte Kontakte benachrichtigt. Diese Idee einer „zentralen“ App wurde jedoch wieder verworfen, nachdem mehrere Datenschutz-Organisationen sie in einen offenen Brief als nicht datenschutzkonform kritisierten (Zentrum für digitalen Fortschritt et al. 2020) und Apple und Google, mit deren Smartphone-Betriebssystemen die App für eine gute Funktionsfähigkeit kompatibel sein sollte, auf einen „dezentralen“ Ansatz bestanden (Floridi 2020). Bei dezentralen Apps werden weniger personenbezogene Daten in einem zentralen Server gespeichert, weshalb sie aus Sicht vieler Datenschützer zu bevorzugen sind (Troncoso et al. 2020).

Die Bundesregierung schwenkte daraufhin auf eine dezentrale App um, die seit dem 16. Juni zum Download bereitsteht. „Sie herunterzuladen und zu nutzen, ist ein kleiner Schritt für jeden von uns, aber ein großer Schritt für die Pandemiebekämpfung. Dabei können die Bürgerinnen und Bürger auf höchsten Datenschutzstandard mit größter Datensparsamkeit vertrauen“, so Kanzleramtschef Helge Braun bei der Vorstellung der App (Bundesregierung 2020). Auf die großen Worte folgten jedoch ernüchternde Ergebnisse. Zwar macht der dezentrale Ansatz genaue Angaben darüber, wie viele Menschen bisher durch die App gewarnt wurden, nicht möglich (Robert Koch Institut 2020a), aber es gibt Daten zur Anzahl positiver Tests, die über die App geteilt wurden. So wurden im Zeitraum von Anfang September 2020 bis Mitte Februar 2021 rund 414.000 positive Testergebnisse via QR-Code oder teleTAN verifiziert, also potenziell per App teilbar gemacht, und von diesen Testergebnissen wurden mit 246.000 knapp 60 % auch tatsächlich geteilt (Robert Koch Institut 2021a). Im gleichen Zeitraum sind aber fast zehn Mal so viele Menschen positiv auf COVID-19 getestet worden (2.263.588 seit der 36. Kalenderwoche 2020, s. Robert Koch Institut 2021b). Angesichts solcher Zahlen kann die Wirksamkeit der App bezweifelt werden, was sich auch in Teilen der öffentlichen Wahrnehmung niederzuschlagen scheint. So war der am häufigsten genannte Grund für den Verzicht auf die Nutzung der App einer Umfrage vom Dezember 2020 zufolge, dass „die App bei der Pandemie-Bekämpfung nicht hilft/… die App nichts bringt“ (Kantar 2020).

Mit digitaler Kontaktverfolgung gegen die Pandemie:^1^ eine Idee, auf der anfangs große Hoffnungen ruhten, ist im Ergebnis enttäuschend. Hätte die digitale Kontaktverfolgung wirksamer gestaltet werden können, und was würde dies für den Datenschutz von Nutzern bedeuten?

In diesem Essay zeigen wir auf, dass die digitale Kontaktverfolgung ein Spannungsfeld zwischen der Datensparsamkeit und einer wirksamen Pandemiebekämpfung erzeugt. Eine umfassende ethische Abwägung ist daher notwendig für die Entscheidung, auf welche digitalen Maßnahmen gesetzt werden sollte. Zum Beispiel greift das von Datenschützern häufig vorgebrachte Argument, dass eine dezentrale Kontaktverfolgung eindeutig zu bevorzugen sei, weil sie sparsamer mit personenbezogenen Daten umgehe, zu kurz, wenn die zusätzlich gesammelten Daten eine deutlich effektivere Pandemiebekämpfung ermöglichen könnte. Wir zeigen, dass dies in der Tat der Fall ist, da zentrale Systeme das Potenzial haben, Infektionsketten schneller zu unterbrechen als dezentrale Systeme, was für die Pandemiebekämpfung einen deutlichen Vorteil bedeutet. Weiterhin argumentieren wir, dass das Spannungsfeld zwischen Datensparsamkeit und einer wirksamen Pandemiebekämpfung sich jedoch nicht in einen Wertekonflikt zwischen Datenschutz und der Wirksamkeit der digitalen Kontaktverfolgung übersetzt. Denn es ist trotz der erhöhten Wirksamkeit nicht der Fall, dass zentrale Systeme uns vor deutlich gravierendere Probleme beim Datenschutz stellen als dezentrale. Zentrale Möglichkeiten der digitalen Kontaktverfolgung sind daher ethisch gerechtfertigt, um auf mögliche weitere Wellen von COVID-19, oder auf zukünftige Infektionskrankheiten, effektiv zu reagieren.^2^

Im nächsten Abschnitt skizzieren wir die Möglichkeiten digitaler Kontaktverfolgung und argumentieren im darauffolgenden Abschnitt, dass der zentrale Ansatz durch die Daten, die hier zusätzlich gesammelt werden, potenziell wirksamer ist als der dezentrale Ansatz. Im darauffolgenden Abschnitt prüfen wir die Argumente gegen den zentralen Ansatz und zeigen, dass diese gegenüber dessen zusätzlicher Wirksamkeit als schwach anzusehen sind. Abschließend betrachten wir im letzten Abschnitt die Konsequenzen unserer Argumente für eine ethisch gerechtfertigte Pandemiebekämpfung.

## Digitale Kontaktverfolgung zur Vermeidung weiterer COVID-19 Wellen

Die vielleicht größte Herausforderung der Pandemiebekämpfung ist es, zwei Szenarien zu vermeiden: eine unkontrollierte Verbreitung des Virus auf der einen Seite und Ausgangsbeschränkungen, die weite Teile der Bevölkerung betreffen („Lockdowns“), auf der anderen. Keines der beiden Szenarien ist wünschenswert: Eine unkontrollierte Verbreitung des Virus würde zu einer hohen Zahl an Todesopfern führen und viele Gesundheitssysteme überlasten (Ferguson et al. 2020), während Ausgangsbeschränkungen verbunden sind mit erheblichen psychologischen, sozialen und wirtschaftlichen Kosten, die darüber hinaus überproportional von historisch benachteiligten Gruppen getragen werden (Fawcett Society et al. 2020) und ihrerseits schwerwiegende Folgen für die öffentliche Gesundheit haben (Savulescu und Cameron 2020). Leichtere Maßnahmen wie das Tragen von Stoffmasken sind hilfreich, aber allein nicht ausreichend, um weitere allgemeine Ausgangsbeschränkungen infolge zweiter COVID-19 Wellen zu verhindern (IHME 2020).

Eine wirksame Kontaktverfolgung verspricht, einen wünschenswerten Mittelweg zwischen den beiden extremen Szenarios zu ebnen: Sie könnte Personen ausfindig machen, die in Kontakt mit Infizierten waren und mit einer hohen Wahrscheinlichkeit ebenfalls infiziert sind, damit diese sich in Quarantäne begeben können. Auf diese Weise könnten Infektionsketten unterbrochen und die Ausbreitung des Virus eingedämmt werden, ohne die Bewegungsfreiheit der allgemeinen Bevölkerung einzuschränken. Die manuelle Kontaktverfolgung, in der Infizierte durch Gesundheitsämter über ihre Kontakte befragt werden, die dann in Kenntnis gesetzt und je nach Umständen unter Quarantäne gestellt werden, ist aber allein nicht ausreichend. Dies liegt daran, dass eine sehr schnelle Unterbrechung von Infektionsketten notwendig ist, um infizierte Personen daran zu hindern, das Virus weiterzugeben (Braithwaite et al. 2020; Ferretti et al. 2020; Kretzschmar et al. 2020). Dies ist besonders wichtig bei SARS-CoV‑2, weil das Virus bereits kurz nach einer Infektion weitergegeben wird (Ganyani et al. 2020; He et al. 2020).

Warn-Apps auf Mobiltelefonen sind dagegen imstande, die Kontaktverfolgung deutlich zu beschleunigen, da sie automatisch Kontakte aufzeichnen und diese im Falle einer wahrscheinlichen Infektion anweisen können, sich testen zu lassen und sich bei positivem Testresultat in Quarantäne zu begeben. Ob eine solche digitale Kontaktverfolgung effektiv ist, hängt wesentlich von zwei Faktoren ab:^3^ Erstens muss sie hinreichend *sensitiv* sein (nicht zu viele COVID-positive Personen auslassen), zweitens muss sie hinreichend *spezifisch* sein (nicht zu viele COVID-negative Personen warnen). Wenn zu wenige Personen gewarnt werden – das System also nicht hinreichend sensitiv ist –, dämmt die App die Verbreitung des Virus nicht wirksam ein. Werden dagegen zu viele Personen gewarnt und unter Quarantäne gestellt – ist das System also nicht hinreichend spezifisch –, würde die Akzeptanz der App verloren gehen. Wir beschreiben im Folgenden den zentralen und den dezentralen Ansatz der digitalen Kontaktverfolgung und betrachten im nächsten Abschnitt, wie die Unterschiede zwischen diesen Ansätzen die Erfüllung dieser Bedingungen beeinflussen kann.

In zentralen wie in dezentralen Systemen tauschen Mobiltelefone über Bluetooth Kennungen aus, wenn sich Nutzer mit aktivierten Apps eine Zeit lang in einer gewissen Nähe zueinander befinden. In einer *dezentralen* App werden diese Kennungen auf den Mobiltelefonen in regelmäßigen Abständen zufällig generiert („zufällige kurzlebige Kennungen“). Wenn eine Person COVID-positiv getestet wird und die Erkrankung über die App meldet, werden ihre seit der Erkrankung generierten Kennungen auf einen zentralen Server geladen (nicht aber die Kennungen ihrer Kontaktpersonen). Weil die App regelmäßig die auf den Server geladenen Kennungen abfragt und mit den ausgetauschten Kennungen abgleicht, können Kontaktpersonen nun automatisch darüber informiert werden, dass sie Kontakt mit einer COVID-positiv getesteten Person hatten, und können sich in Quarantäne begeben.

In einer *zentralen* App erhält dagegen jeder Benutzer eine permanente pseudonyme Kennung, die auf einem zentralen Server gespeichert ist. Der Server generiert auch zufällige kurzlebige Kennungen, die Mobiltelefonen zugespielt werden (statt dass Mobiltelefone diese Kennungen selbst generieren, wie in der dezentralen Variante). Wenn eine Person ihre Erkrankung meldet, lädt ihr Telefon die kurzlebigen Kennungen ihrer Kontaktpersonen auf den Server, wo das Risiko der Begegnung und die pseudonymen Kennungen der Kontaktpersonen ermittelt werden. Wie in der dezentralen Variante können Kontaktpersonen dann automatisch darüber informiert werden, dass sie Kontakt mit einer COVID-positiv getesteten Person hatten.

Befürworter des dezentralen Ansatzes monieren, dass das zentrale Speichern von permanenten Kennungen und Informationen über Kontaktpersonen es Behörden wie Gesundheitsämtern, aber möglicherweise auch Hackern, erlauben würde, Nutzer zu identifizieren. Sie sind auch besorgt über eine mögliche schleichende Ausweitung der Überwachung und die Nutzung von Daten für andere Zwecke als die Seuchenbekämpfung, z. B. die Strafverfolgung (Joint Statement 2020; Lomas 2020). Sie argumentieren, dass es nicht nötig sei, diese zusätzlichen Daten zu sammeln und zu speichern, und dass daher der dezentrale Ansatz zu bevorzugen sei (Joint Statement 2020; Lomas 2020; Troncoso et al. 2020). Zudem legen sie nahe, dass die Technologie nicht angenommen und in hinreichend großen Zahlen installiert werden würde, falls die Daten nicht durch einen dezentralen Ansatz geschützt sind (Europäisches Parlament 2020; Joint Statement 2020; Troncoso et al. 2020). Die Technologieunternehmen Google und Apple scheinen diese Überzeugungen zu teilen und setzen, wie eingangs erwähnt, auf dezentrale Apps: Die beiden tech-Giganten haben eine Anwendungsschnittstelle entwickelt, die den zuverlässigen Austausch von Kennungen über Bluetooth über ihre Betriebssysteme ermöglicht – aber nur, wenn die Anwendung dezentral ist.

## Warum der zentrale Ansatz eine wirksamere Kontaktverfolgung ermöglicht

Das Argument, dass der dezentrale Ansatz zu bevorzugen sei, weil er weniger Daten zentral speichert und damit sparsamer mit Daten von Nutzern umgehe, ignoriert die Frage, ob die Daten, die im zentralen Ansatz zusätzlich gesammelt werden, eine wirksamere Kontaktverfolgung erlauben könnten (d. h. die Bedingungen der Sensitivität oder Spezifität besser erfüllen). Wir argumentieren im Folgenden, dass diese Frage mit „ja“ beantwortet werden kann. Damit stellt die digitale Kontaktverfolgung uns vor ethische Probleme, die nicht vom Prinzip abgedeckt sind, dass von zwei *vergleichbar wirksamen* Ansätzen der datensparsamere zu bevorzugen sei. Der Grund für die potenziell bessere Wirksamkeit ist, dass falsch-positive Meldungen ein Problem für dezentrale Systeme darstellen, weniger dagegen für zentrale Systeme. Deshalb könnte ein zentrales System die zwei Bedingungen der Sensitivität und Spezifität besser erfüllen als ein dezentrales. Im Folgenden erklären wir dieses Problem im Detail.

Eine zentrale Frage für die Erfüllung der Spezifität eines Systems ist, wie sichergestellt werden kann, dass die Infektionsmeldungen über eine App korrekt sind. Die einfachste und schnellste Möglichkeit wäre, dass Nutzer sich selbst positiv melden können, sobald sie Symptome von COVID-19 haben. Jedoch würden viele Systeme dann mit falsch-positiven Meldungen überlaufen (Sweeney 2020). Solche falsch-positiven Meldungen würden unvermeidbar getätigt werden, zum Beispiel, weil Symptome fälschlich für eine COVID-19 Erkrankung gehalten werden, oder wegen missbräuchlicher Anwendung. Je mehr falsch-positive Meldungen getätigt werden, desto mehr vermeintliche Risikokontakte werden fälschlicherweise aufgefordert, sich in Quarantäne zu begeben, sodass das System nicht hinreichend spezifisch und seine Wirksamkeit damit eingeschränkt ist.

Um das Problem der falsch-positiven Meldungen zu umgehen, erlaubt die deutsche Corona-Warn-App eine Meldung nur bei Vorliegen eines positiven PCR Testresultats, das Nutzer über eine teleTAN oder einen QR-Code in der App registrieren lassen können (Robert Koch Institut 2020a). Auf diese Weise werden viele falsch-positive Meldungen unterbunden, aber die Kontaktverfolgung wird gleichzeitig deutlich verlangsamt, weil Kontakte von Infizierten erst bei Vorliegen von deren positiven Tests gewarnt werden.^4^ In der Zeit vor der Warnung können diese Kontakte aber bereits weitere Menschen infiziert haben. In der Tat legen mathematische Modelle nahe, dass schon Verzögerungen von einem halben Tag zwischen Einsetzen der Symptome und Warnung der Kontakte den Unterschied machen können zwischen erfolgreicher Pandemiebekämpfung und einer erneuten unkontrollierten Ausbreitung (Hinch et al. 2020).

In einer zentralen App könnte dieses Problem dagegen ohne Zeitverzögerung umgangen werden. Denn Nutzer könnten sich als infiziert melden, sobald sie Symptome zeigen oder ein positiver Schnelltest vorliegt und ohne auf einen positiven PCR Testbefund zu warten. Die Kontakte dieser Personen würden dann zwar aufgefordert, sich in Quarantäne zu begeben. Der Unterschied zu einer dezentralen Variante ist aber, dass sie sofort wieder aus der Quarantäne entlassen werden könnten, falls die Meldung sich als falsch herausstellt. Zum Beispiel könnte eine Person mit Symptomen sich nach ihrer Meldung testen lassen und bei negativem Testergebnis dies der App melden, woraufhin die Kontakte rasch benachrichtigt und aus ihrer Quarantäne entlassen werden könnten.

Warum ist dies nicht möglich in dezentralen Systemen? Abgesehen von kurzlebigen Kennungen bei Meldung einer Infektion werden beim dezentralen Ansatz keine Informationen über Nutzer auf einem zentralen Server gespeichert, weshalb ein Zugang zum zentralen Server keine Rückschlüsse über Nutzer zulässt. Theoretisch könnte man auch im dezentralen Ansatz ermöglichen, dass jemand mit Symptomen dies in der App meldet, aber diese Meldung nach negativem Testergebnis zurückzieht, worüber die Kontakte benachrichtigt und aus ihrer Quarantäne entlassen würden. Das Problem ist aber, dass Fälle, bei denen nach solcher Meldung kein Testergebnis in der App registriert wird (etwa weil kein Test gemacht wurde, oder Nutzer vergessen, das Testergebnis in der App zu registrieren), nicht identifizierbar sind. Ein zentraler Ansatz könnte dagegen diese falsch-positiven Meldungen einfangen. Zum Beispiel könnten nach einer bestimmten Zeitspanne diejenigen Cluster aus der Quarantäne entlassen werden, die wahrscheinlich auf einer falschen positiven Meldung beruhen, wenn keine oder nur wenige Kontakte der Nutzerin oder des Nutzers, die oder der die Meldung getätigt hat, Symptome zeigen (Hinch et al. 2020). Dieser Prozess funktioniert nur in zentralen Systemen, wo der Server über permanente pseudonyme Kennungen von Nutzern verfügt und daher nachvollzogen werden kann, wer mit wem Kontakt hatte. Dies bedeutet im Übrigen nicht, dass individuelle Nutzer identifiziert werden, da das Nachvollziehen von Kontakten auf Basis von *pseudonymen* Kennungen geschieht (Vaudenay 2020).^5^

Wir weisen darauf hin, dass das hier vorgestellte zentrale System auf Grundlagenforschung beruht (Ferretti et al. 2020; Hinch et al. 2020). Es unterscheidet sich von zentralen Systemen, die bisher implementiert wurden, wie der französischen App, die einen positiven Test vor einer Meldung verlangt, und in der Praxis könnte sein Design und Implementierung Probleme aufwerfen, z. B. wie die missbräuchliche Anwendung ausgeschlossen werden kann.^6^ Dennoch zeigt sich ein klarer Vorteil gegenüber dezentralen Systemen darin, dass zentrale Systeme potenziell Meldungen ohne Zeitverzögerung erlauben könnten (s. auch White und van Basshuysen 2021a). Darüber hinaus haben die zusätzlichen Daten, die in einem zentralen System gesammelt werden, noch weitere Vorteile, weil sie für die Kalibrierung des Systems genutzt werden könnten. So kann von vergangenen Fällen, je nachdem ob eine Infektion stattfand oder nicht, gelernt werden, welche Arten von Kontakten (Zeitraum, Nähe des Kontakts etc.) zu hohen Übertragungsrisiken führen und welche nicht (Hinch et al. 2020). Solche Informationen könnten dafür benutzt werden, Quarantänebestimmungen spezifisch an das individuelle Infektionsrisiko anzupassen (Ferretti et al. 2020). Darüber hinaus könnten neben Kontaktpersonen von positiv gemeldeten Nutzern auch Zweitkontakte gewarnt werden, zum Beispiel eine Person, die in Kontakt war mit jemandem, der wiederum Kontakt zu einer Person hatte, die nun COVID-19 Symptome über die App meldet. Dieses Vorgehen könnte die Kontaktverfolgung weiter beschleunigen (Ferretti et al. 2020; Hinch et al. 2020).

Aufgrund der potenziell wirksameren Kontaktverfolgung durch eine zentrale App sollte das von vielen Datenschützern vorgebrachte Argument, dass es nicht nötig sei, diese zusätzlichen pseudonymisierten Daten zu sammeln und zu speichern, und dass daher der dezentrale Ansatz zu bevorzugen sei, neu überdacht werden. Denn mit den zusätzlichen Daten ist es wahrscheinlicher als ohne, dass die beiden Bedingungen für erfolgreiche Kontaktverfolgung – Spezifität und Sensitivität – hinreichend erfüllt werden können. Falls es aus Sicht der Datenschutz-Ethik problematisch ist, diese zusätzlichen Daten zu sammeln und zu speichern, haben wir einen Wertekonflikt, weil wir uns zwischen dem Datenschutz und der Wirksamkeit digitaler Pandemiebekämpfung entscheiden müssten und mit dieser Entscheidung wahrscheinlich einen Wert verletzen würden: den Schutz der Privatsphäre, oder die Rettung von Menschenleben vor dem Virus. Betrachten wir also, ob es tatsächlich datenschutzethisch problematisch ist, diese zusätzlichen Daten zu sammeln und zu speichern.

## Ist der zentrale Ansatz aus Sicht der Datenschutz-Ethik problematisch?

Befürworter des dezentralen Ansatzes sehen die zusätzlichen Daten, die in zentralen Ansätzen gesammelt werden, nicht nur als unnötig an, um die Ziele der App zu erreichen (eine Behauptung, gegen die wir im letzten Abschnitt argumentiert haben), sondern sorgen sich auch um mögliche Probleme, die die zentrale Speicherung dieser Daten mit sich bringen. Sie stützen diese Bedenken vor allem auf drei mögliche Szenarien: Erstens könnten Regierungen aufgrund dieser Daten – obwohl pseudonym gespeichert – Nutzer einer zentralen App identifizieren und diese Informationen möglicherweise für andere Zwecke, wie die Strafverfolgung, missbrauchen (Joint Statement 2020; Lomas 2020; Troncoso et al. 2020); zweitens wäre es möglich, dass die Daten auf dem zentralen Server Opfer eines Hackerangriffs werden (Joint Statement 2020); und drittens könnte ein zentrales System, gerade weil es anfällig ist für Missbrauch der gespeicherten Daten, dazu führen, dass Bürger der App nicht vertrauen und sie deshalb nicht von einem hinreichend großen Prozentsatz der Bevölkerung benutzt wird (Europäisches Parlament 2020; Joint Statement 2020; Troncoso et al. 2020).

Betrachten wir die möglichen Szenarien im Einzelnen. Wir stimmen mit Datenschützern überein, dass die Möglichkeit, dass persönliche Daten aus der Kontaktverfolgung für andere Zwecke missbraucht werden, als unzulässig angesehen werden muss. Jedoch folgt daraus nicht zwingend, dass eine zentrale Speicherung pseudonymisierter Daten unzulässig ist. Wenn es möglich wäre, den zentralen Server vor missbräuchlichem Zugriff auf seine Daten durch Regierungsbehörden zu schützen, so würde die potenziell höhere Wirksamkeit zentraler Systeme einen Grund darstellen, diese zu bevorzugen. In Ländern wie Deutschland könnte solch ein Schutz gewährleistet werden. Die Voraussetzung wäre eine Gesetzgebung, die den staatlichen Zugriff auf den zentralen Server klar regelt und insbesondere die sachfremde Nutzung von Informationen ausschließt. In einigen Ländern gibt es solch eine Gesetzgebung bereits; so haben einige US-Staaten Gesetze eingeführt, die *unter keinen Bedingungen* das Benutzen von Daten, die aus der COVID-19 Kontaktverfolgung entstanden sind, für sachfremde Zwecke erlauben (z. B. New York State Senat 2020). Ähnliche Gesetze haben bereits in anderen Epidemien einen möglichen Missbrauch der Daten von erkrankten Personen unterbunden. Zum Beispiel wurde im kanadischen British Columbia die Weitergabe von Daten von HIV-positiv getesteten Personen zum Zwecke der Strafverfolgung ausgeschlossen, mit der Begründung, dass „die angeordnete Weitergabe vertraulicher Informationen die Möglichkeit einer wirksamen HIV-Behandlung unterbindet und das Leben von HIV-positiven Personen gefährdet, was wiederum ein Risiko für die Gesundheits-Interessen der Gesamtbevölkerung darstellen würde“ (Provincial Court of British Columbia 2014; Übersetzung der Autoren). Der Punkt hier ist, dass es möglich ist, die Weitergabe von Daten aus der Kontaktverfolgung zu regulieren, um die Zwecke, zu denen diese Daten benutzt werden können, klar zu begrenzen.^7^

Betrachten wir nun das zweite problematische Szenario für den zentralen Ansatz, nämlich, dass die Daten auf dem zentralen Server Opfer eines Hackerangriffs werden könnten. Mögliche Angriffe bergen jedoch auch Probleme für den dezentralen Ansatz. Zum Beispiel könnten böswillige Nutzer in dezentralen Apps die Identitäten anderer Nutzer aufdecken, die sich als infiziert gemeldet haben: Da alle kurzlebigen Kennungen dieser Nutzer auf den zentralen Server geladen werden, könnten sie von einem Nutzer identifiziert werden, der davor deren kurzlebige Kennungen aufgezeichnet hat (Ahmed et al. 2020; Vaudenay 2020). Zentrale Systeme sind gegen solche Angriffe besser geschützt, und Angriffe gegen diese Systeme können einfacher erkannt werden (Vaudenay 2020). Dagegen sind erfolgreiche Angriffe, wenn auch weniger wahrscheinlich, in zentralen Systemen potenziell schwerwiegender, weil ein Hacker möglicherweise Zugriff auf mehr Nutzerdaten der App erhalten und Nutzer durch ihre zentral gespeicherten Kennungen identifizieren könnte (White und van Basshuysen 2021a, 2021b; vgl. auch Baskerville et al. 2018).^8^ Verschiedene Systeme bergen also verschiedene Arten von Risiken, aber es ist nicht der Fall, dass dezentrale Systeme „durch Design“ die Privatsphäre wahren, wie manchmal von Datenschützern behauptet wird (Joint Statement 2020). Das Risiko von Hackerangriffen stellt damit kein Argument für dezentrale Systeme dar, weil diese hier keineswegs klar im Vorteil gegenüber zentralen Systemen sind. Vielmehr sollte in der Bewertung dieser Risiken die potenzielle Wirksamkeit eines Systems miteinbezogen werden: Wenn ein System nur eine geringe Chance hat, eine wirksame Kontaktverfolgung zu ermöglichen, so ist dies ein Grund, die zugehörigen Risiken sehr behutsam abzuwägen; wenn dagegen eine wirksame Kontaktverfolgung wahrscheinlicher ist, kann das zugehörige Risiko, das wir bereit sein sollten zu akzeptieren, möglicherweise höher sein (White und van Basshuysen 2021a, 2021b).

Schließlich das dritte mögliche Problem, nämlich, dass ein zentrales System, da anfällig für Missbrauch, nicht von einem hinreichend großen Prozentsatz der Bevölkerung heruntergeladen und benutzt wird. Aber die Anfälligkeit für die Identifizierung von infizierten Nutzern legt nahe, dass dieses Problem nicht nur zentrale, sondern auch dezentrale Systeme beeinträchtigen könnte. In der Tat hat in einer empirischen Studie in den USA die Mehrheit der Teilnehmer angegeben, eher eine zentrale als eine dezentrale App zu installieren, weil sie eher bereit seien das Risiko einzugehen, dass eine zentrale Behörde Zugriff auf Identitäten von Nutzern erhält, als anderen Nutzern Rückschlüsse über die Identität von Infizierten zu ermöglichen (Li et al. 2020). Wenn die digitale Kontaktverfolgung über eine freiwillige App erfolgt, besteht immer die Gefahr, dass die App für eine effektive Kontaktverfolgung nicht hinreichend oft heruntergeladen wird, was aber durch Anreize – zum Beispiel mobiles Guthaben für die Nutzung der App – wahrscheinlicher gemacht werden könnte (Loi 2020). Dies ist aber kein Problem, das nur zentrale Systeme betrifft, und könnte möglicherweise ein größeres Problem für dezentrale Systeme darstellen.

Zusammenfassend bergen sowohl zentrale als auch dezentrale Systeme aus Sicht der Datenschutz-Ethik potenzielle Probleme, weshalb die Implementierung eines Systems gesetzlich klar geregelt werden und Datenschutz-Experten bei dessen Design hinzugezogen werden sollten. Es ist aber unwahrscheinlich, dass diese Probleme bei zentralen Systemen viel gravierender sind als bei dezentralen, und das Problem der Identifizierung von infizierten Nutzern bei dezentralen Systemen legt nahe, dass das Gegenteil der Fall sein könnte. Aus dieser Beobachtung und der größeren möglichen Wirksamkeit der zentralen digitalen Kontaktverfolgung folgt, dass, wer auf digitale Kontaktverfolgung setzt, einem zentralen System den Vorzug geben sollte.

## Implikationen für eine ethische digitale Pandemiebekämpfung

Kommen wir zurück zum anfangs gemachten Rückblick. Was ging schief auf dem Weg zu vermeintlich besseren, weil datensparsameren, Lösungen der digitalen Kontaktverfolgung, der am Ende im Design einer Corona-Warn-App mündete, die im Ergebnis weitgehend wirkungslos ist? Julian Nida-Rümelin hat dies treffend formuliert, als er im öffentlichen Fernsehen über die deutsche Strategie zur Pandemiebekämpfung kritisch anmerkte: „Wir verletzen im Grunde alle Grundrechte … und dann sagen wir, aber informationelle Selbstbestimmung ist so hoch zu halten, dass wir lieber einen Lockdown machen, als in dem Punkt Einschränkungen“ (DasErste 2020).

Der Aspekt der Datensparsamkeit hat öffentliche und politische Debatten zunehmend dominiert auf Kosten von anderen ethischen Gesichtspunkten wie der möglichen Einschränkung der Wirksamkeit der Kontaktverfolgung, die in diesen Debatten immer mehr ignoriert wurden. Wie wir gezeigt haben, können aber zusätzliche, zentral gespeicherte Daten die digitale Kontaktverfolgung beschleunigen und damit ihre Wirksamkeit erhöhen. Wenn diese Daten pseudonymisiert sind und ihre Nutzung gesetzlich klar geregelt ist, ist eine missbräuchliche Anwendung nicht wahrscheinlicher als bei dezentralen Lösungen. Aus diesen Gründen lässt das Spannungsfeld zwischen Datensparsamkeit und effektiver Pandemiebekämpfung sich nicht in einen Wertekonflikt zwischen Datenschutz und Effektivität übersetzen, sondern der Aspekt der Wirksamkeit überwiegt deutlich. Zentrale Lösungen würden deshalb eine ethisch besser gerechtfertigte digitale Pandemiebekämpfung erlauben. Diese Schlussfolgerung sollte Anstoß geben für eine erneute Debatte darüber, wie das Potenzial der digitalen Kontaktverfolgung besser ausgeschöpft werden kann, um auf weitere Wellen von COVID-19 oder auf zukünftige Epidemien zu reagieren.

## Footnotes

1 Wir sprechen von digitaler Kontaktverfolgung, weil sich dieser Begriff für Corona-Warn-Apps eingebürgert hat. Die deutsche „Corona Warn App“ ermöglicht keine Kontaktverfolgung im engen Sinn, da sie keine Infektionsketten nachvollzieht, sondern ihr Ziel ist es, Personen zu warnen, die sich in der Nähe von positiv getesteten Personen aufgehalten haben (s. Troncoso et al. 2020). Wir erklären in den nächsten Abschnitten, dass anders konstruierte Systeme es erlauben würden, begrenzte Informationen über Infektionsketten zu sammeln, und wie dies die Wirksamkeit der Systeme verbessern könnte.

2 Dass wir uns in der Diskussion auf die Datensparsamkeit und die Wirksamkeit digitaler Kontaktverfolgung konzentrieren, bedeutet nicht, dass andere Werte für Design und Implementierung digitaler Systeme nicht wichtig sind (dazu s. Gasser et al. 2020; Mello und Wang 2020; Morley et al. 2020; Ranisch et al. 2020). Die meisten dieser Werte erzeugen aber keine vergleichbaren Zielkonflikte (z. B. kann Transparenz erreicht werden, ohne dass andere wichtige Werte verletzt werden), weshalb eine Abwägung von Datensparsamkeit und Wirksamkeit für die Ethik der digitalen Kontaktverfolgung zentral ist.

3 Um wirksam zu sein, müssen darüber hinaus weitere Bedingungen erfüllt sein, insbesondere hinreichende Testkapazitäten und Penetrationsraten von Smartphones, sowie die gesellschaftliche Akzeptanz der Apps. Daneben könnte die Integration weiterer Funktionen, z. B. die Clustererkennung oder ein Kontakttagebuch, die Wirksamkeit der Apps erhöhen. Wir danken einem/r anonymen Gutachter/in für diese Hinweise.

4 Eine breite Verfügbarkeit von Schnelltests könnte dieses Problem möglicherweise lindern, wenn man sich auf Basis eines solchen Tests in der App als positiv melden könnte. Schnelltests können das Problem aber nicht gänzlich beheben, weil sie eine geringe Sensitivität aufweisen (70–75 %) (Corman et al. 2020; Krüger et al. 2020) und in Deutschland nach wie vor ein Mangel und Probleme bei der Verteilung vorherrschen (Tagesschau 2021). Wenn Schnelltests in hinreichender Zahl vorhanden sein sollten, könnten diese aber komplementär zur zentralen Kontaktverfolgung benutzt werden – Schnelltest-Resultate könnten ohne Zeitverzögerung in der App registriert werden, und könnten daraufhin dahingehend überprüft werden, ob Cluster identifiziert werden (s. unten). Dies würde sowohl die Anzahl an Nutzern, die fälschlicherweise gewarnt würden, als auch die Länge des Bestehens einer fälschlichen Warnung verringern.

5 Manche Datenschützer argumentieren jedoch, dass es Regierungen möglich wäre, mithilfe dieser pseudonymen Kennungen Nutzer zu identifizieren und diese Informationen für sachfremde Zwecke wie die Strafverfolgung zu benutzen; für Diskussionen dieser Argumente s. White und van Basshuysen (2021b).

6 Eine weitere praktische Herausforderung wäre die Frage, wie eine funktionale App ohne die oben erwähnte, von Apple und Google entwickelte Schnittstelle, entworfen werden kann. Hier sei nur erwähnt, dass die französische, zentrale App ohne die Schnittstelle auskommt.

7 Unser Argument ist beschränkt auf Länder wie Deutschland, in denen die Nutzung von Daten reguliert ist und diese Regulierung auch adäquat umgesetzt werden kann, inklusive einer Gerichtsbarkeit, die einen Missbrauch von staatlicher Seite auch rückwirkend unwahrscheinlich macht. In einigen Ländern ist dies jedoch nicht der Fall. Hier könnte den Herstellern von Betriebssystemen eine tragende Rolle zukommen, indem sie Länder, in denen Daten für andere Zwecke missbraucht werden, von der Kontaktverfolgung über Mobiltelefone ausschließen. Es ist problematisch, wenn Privatunternehmen auf diese Weise politische Entscheidungen beeinflussen können (Sharon 2020), geschieht de facto aber schon in der gegenwärtigen Situation, in der Apple und Google über gewählte Regierungen hinweg entschieden haben, nur dezentrale Apps zu unterstützen (Floridi 2020).

8 Wir danken einem/r anonymen Gutachter/in für den Hinweis, dass bei einem erfolgreichen Angriff unterschiedliche Arten und Umfänge von Daten kompromittiert wären.
